# 2-[2-(4-Benzyl­piperazin-1-ylcarbon­yl)eth­yl]-5,6-diphenyl­pyridazin-3(2*H*)-one

**DOI:** 10.1107/S1600536808013159

**Published:** 2008-05-10

**Authors:** Abdullah Aydın, Deniz S. Doğruer, Mehmet Akkurt, Orhan Büyükgüngör

**Affiliations:** a Department of Science Education, Faculty of Education, Kastamonu University, 37200 Kastamonu, Turkey; b Department of Pharmaceutical Chemistry, Faculty of Pharmacy, Gazi University, 06330 Ankara, Turkey; c Department of Physics, Faculty of Arts and Sciences, Erciyes University, 38039 Kayseri, Turkey; d Department of Physics, Faculty of Arts and Sciences, Ondokuz Mayıs University, 55139 Samsun, Turkey

## Abstract

The title compound, C_30_H_30_N_4_O_2_, has a non-planar conformation, the dihedral angles formed by the pyridazinone ring plane and the three phenyl rings being 54.61 (7), 51.10 (7) and 59.53 (8)°. The piperazine ring adopts a chair conformation. Inter- and intra­molecular C—H⋯O contacts are found in the crystal structure and these consolidate the three-dimensional packing.

## Related literature

For related structures, see: Doğruer *et al.* (2007 ▶); Swenson *et al.* (1997 ▶); Yüksektepe *et al.* (2004 ▶). For structure analysis, see: Allen *et al.* (1987 ▶); Cremer & Pople (1975 ▶).
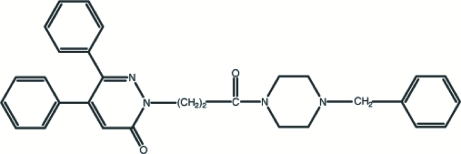


## Experimental

### 

#### Crystal data

C_30_H_30_N_4_O_2_
*M* *_r_* = 478.58
Monoclinic, 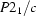

*a* = 15.6725 (12) Å
*b* = 9.1139 (5) Å
*c* = 17.6743 (12) Å
β = 90.553 (6)°
*V* = 2524.4 (3) Å^3^
*Z* = 4
Mo *K*α radiation
μ = 0.08 mm^−1^
*T* = 296 K
0.73 × 0.51 × 0.11 mm

#### Data collection

Stoe IPDS-2 diffractometer
Absorption correction: integration (*X-RED32*; Stoe & Cie, 2002 ▶) *T*_min_ = 0.477, *T*_max_ = 0.907
30083 measured reflections
4966 independent reflections
3394 reflections with *I* > 2σ(*I*)
*R*_int_ = 0.062

#### Refinement

*R*[*F* ^2^ > 2σ(*F* ^2^)] = 0.041
*wR*(*F* ^2^) = 0.094
*S* = 1.03
4966 reflections
325 parameters
H-atom parameters constrained
Δρ_max_ = 0.14 e Å^−3^
Δρ_min_ = −0.15 e Å^−3^

### 

Data collection: *X-AREA* (Stoe & Cie, 2002 ▶); cell refinement: *X-AREA*; data reduction: *X-RED32* (Stoe & Cie, 2002 ▶); program(s) used to solve structure: *SIR97* (Altomare *et al.*, 1999 ▶); program(s) used to refine structure: *SHELXL97* (Sheldrick, 2008 ▶); molecular graphics: *ORTEP-3 for Windows* (Farrugia, 1997 ▶); software used to prepare material for publication: *WinGX* (Farrugia, 1999 ▶).

## Supplementary Material

Crystal structure: contains datablocks global, I. DOI: 10.1107/S1600536808013159/tk2267sup1.cif

Structure factors: contains datablocks I. DOI: 10.1107/S1600536808013159/tk2267Isup2.hkl

Additional supplementary materials:  crystallographic information; 3D view; checkCIF report

## Figures and Tables

**Table 1:** Hydrogen-bond geometry (Å, °)

*D*—H⋯*A*	*D*—H	H⋯*A*	*D*⋯*A*	*D*—H⋯*A*
C2—H2⋯O2^i^	0.93	2.47	3.3470 (19)	157
C18—H18*B*⋯O1	0.97	2.56	3.0755 (18)	113
C20—H20*B*⋯O2	0.97	2.35	2.759 (2)	105

Symmetry code: (i) 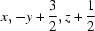.

**Fig. 1.**
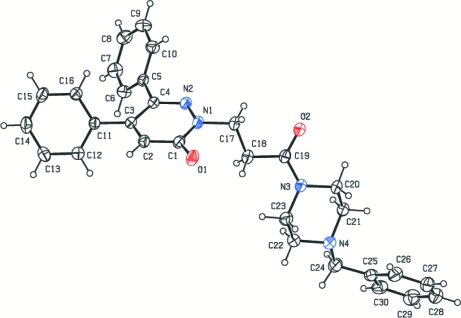
The molecular structure of (I) showing the atom numbering scheme. Displacement ellipsoids for non-H atoms are drawn at the 30% probability level.

**Fig. 2.**
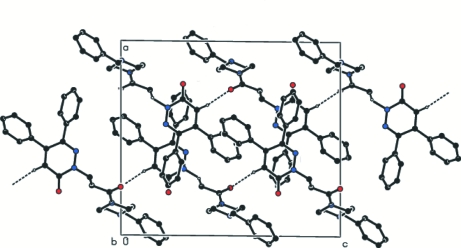
View of the hydrogen bonding interactions (dashed lines) in (I) down b axis. H atoms not involved in hydrogen bonding interactions have been omitted for clarity.

## supplementary crystallographic information

### Comment

The title compound (I) was prepared recently (Doğruer *et al.*, 2007) and displays analgesic and anti-inflammatory effects. In the crystal structure of (I), Fig. 1, the bond lengths and angles are within their normal ranges (Allen *et al.*, 1987). The piperazine bridge has a normal chair conformation, with puckering parameters (Cremer & Pople, 1975) *Q*_T_ = 0.562 (2) Å, θ = 176.2 (2) ° and φ = 354 (3) °. Relevant literature values for the puckering of the cyclobutane ring are 29.0 (1)° (Yüksektepe *et al.*, 2004) and 23.5° (Swenson *et al.*, 1997). In this study, the N1/N2/C1—C4 pyridazinone ring plane forms dihedral angles of 54.61 (7), 51.10 (7) and 59.53 (8)°, respectively, with the planes of the (A: C5—C10), (B: C11—C16) and (C: C25 –C30) phenyl rings. The dihedral angles between the phenyl rings are A/B = 50.77 (8), A/C = 83.01 (9) and B/C = 70.15 (9)°.

The crystal structure is stabilized by inter and intramolecular C—H···O contacts that stabilize the three-dimensional network (Table 1, Fig. 2).

### Experimental

[3-(5,6-Diphenyl-3(2*H*)-pyridazinone-2-yl)propanoic acid (0.01 mol) in dichloromethane (40 ml) at 273 K (ice-bath) was treated with triethylamine (1 ml) and ethyl chloroformate (0.01 mol). After stirring the reaction mixture at 273 K for 15 min, benzylpiperazine (0.011 mol) was added. The final mixture was stirred at 273–298 K for 24 h and evaporated to dryness. The product was solidified with ice-cold water and crystallized from ethanol (yield 25%, m.p. 422 K). IR *v*_max_(cm^-1^) (KBr): 1660 (CO ring), 1635 (CO amide) (Doğruer *et al.*, 2007).

### Refinement

All H atoms were then placed in geometrically idealized positions and constrained to ride on their parent atoms, with C*sp*^3^—H = 0.97 Å and C*sp*^2^—H = 0.93 Å, and with *U*_iso_(H) = 1.2*U*_eq_(C).

### Crystal data

**Table:**

C_30_H_30_N_4_O_2_	*F*_000_ = 1016
*M**_r_* = 478.58	*D*_x_ = 1.259 Mg m^−^^3^
Monoclinic, *P*2_1_/*c*	Mo *K*α radiation λ = 0.71073 Å
Hall symbol: -P 2ybc	Cell parameters from 32998 reflections
*a* = 15.6725 (12) Å	θ = 1.8–28.0º
*b* = 9.1139 (5) Å	µ = 0.08 mm^−^^1^
*c* = 17.6743 (12) Å	*T* = 296 K
β = 90.553 (6)º	Plate, colourless
*V* = 2524.4 (3) Å^3^	0.73 × 0.51 × 0.11 mm
*Z* = 4	

### Data collection

**Table:**

Stoe IPDS-2 diffractometer	4966 independent reflections
Monochromator: plane graphite	3394 reflections with *I* > 2σ(*I*)
Detector resolution: 6.67 pixels mm^-1^	*R*_int_ = 0.062
*T* = 296 K	θ_max_ = 26.0º
rotation method scans	θ_min_ = 2.3º
Absorption correction: integration(XRED32; Stoe & Cie, 2002)	*h* = −19→19
*T*_min_ = 0.477, *T*_max_ = 0.907	*k* = −11→11
30083 measured reflections	*l* = −21→21

### Refinement

**Table:**

Refinement on *F*^2^	Secondary atom site location: difference Fourier map
Least-squares matrix: full	Hydrogen site location: inferred from neighbouring sites
*R*[*F*^2^ > 2σ(*F*^2^)] = 0.041	H-atom parameters constrained
*wR*(*F*^2^) = 0.094	*w* = 1/[σ^2^(*F*_o_^2^) + (0.0452*P*)^2^ + 0.0381*P*] where *P* = (*F*_o_^2^ + 2*F*_c_^2^)/3
*S* = 1.03	(Δ/σ)_max_ < 0.001
4966 reflections	Δρ_max_ = 0.14 e Å^−^^3^
325 parameters	Δρ_min_ = −0.15 e Å^−^^3^
Primary atom site location: structure-invariant direct methods	Extinction correction: none

### Special details

**Table:**

Geometry. Bond distances, angles *etc*. have been calculated using the rounded fractional coordinates. All su's are estimated from the variances of the (full) variance-covariance matrix. The cell e.s.d.'s are taken into account in the estimation of distances, angles and torsion angles
Refinement. Refinement on *F*^2^ for ALL reflections except those flagged by the user for potential systematic errors. Weighted *R*-factors *wR* and all goodnesses of fit *S* are based on *F*^2^, conventional *R*-factors *R* are based on *F*, with *F* set to zero for negative *F*^2^. The observed criterion of *F*^2^ > σ(*F*^2^) is used only for calculating -*R*-factor-obs *etc*. and is not relevant to the choice of reflections for refinement. *R*-factors based on *F*^2^ are statistically about twice as large as those based on *F*, and *R*-factors based on ALL data will be even larger.

### Fractional atomic coordinates and isotropic or equivalent isotropic displacement parameters (Å^2^)

**Table:**

	*x*	*y*	*z*	*U*_iso_*/*U*_eq_	
O1	0.77910 (7)	0.86642 (13)	0.78316 (6)	0.0564 (4)	
O2	0.75320 (8)	0.76212 (12)	0.50119 (6)	0.0545 (4)	
N1	0.65740 (8)	0.85831 (14)	0.71319 (7)	0.0435 (4)	
N2	0.57156 (8)	0.85469 (13)	0.70467 (7)	0.0429 (4)	
N3	0.83628 (9)	0.57588 (14)	0.54110 (7)	0.0483 (4)	
N4	0.89677 (9)	0.28978 (14)	0.50320 (7)	0.0512 (5)	
C1	0.70100 (10)	0.85822 (16)	0.78161 (8)	0.0430 (5)	
C2	0.64657 (10)	0.84702 (16)	0.84615 (8)	0.0429 (5)	
C3	0.56060 (9)	0.84191 (14)	0.84000 (8)	0.0380 (4)	
C4	0.52408 (9)	0.84847 (15)	0.76493 (8)	0.0378 (4)	
C5	0.43077 (9)	0.84729 (15)	0.74879 (8)	0.0383 (4)	
C6	0.37801 (11)	0.73728 (17)	0.77581 (9)	0.0490 (5)	
C7	0.29174 (12)	0.73806 (19)	0.75865 (10)	0.0568 (6)	
C8	0.25695 (11)	0.84874 (19)	0.71553 (10)	0.0568 (6)	
C9	0.30880 (12)	0.95805 (19)	0.68845 (10)	0.0570 (6)	
C10	0.39510 (11)	0.95668 (17)	0.70496 (9)	0.0481 (5)	
C11	0.50675 (9)	0.82763 (15)	0.90846 (8)	0.0391 (4)	
C12	0.52513 (11)	0.72082 (17)	0.96183 (9)	0.0498 (5)	
C13	0.47574 (13)	0.7070 (2)	1.02585 (9)	0.0591 (6)	
C14	0.40832 (12)	0.8005 (2)	1.03718 (10)	0.0605 (6)	
C15	0.39035 (11)	0.90860 (19)	0.98546 (9)	0.0538 (6)	
C16	0.43914 (10)	0.92255 (17)	0.92135 (8)	0.0454 (5)	
C17	0.70489 (11)	0.85361 (17)	0.64203 (8)	0.0490 (5)	
C18	0.74754 (11)	0.70578 (16)	0.63244 (8)	0.0481 (5)	
C19	0.78017 (10)	0.68481 (16)	0.55269 (8)	0.0410 (5)	
C20	0.86743 (11)	0.54334 (17)	0.46501 (8)	0.0486 (5)	
C21	0.85466 (11)	0.38316 (17)	0.44691 (9)	0.0485 (5)	
C22	0.86159 (14)	0.32222 (19)	0.57767 (10)	0.0624 (6)	
C23	0.87401 (13)	0.4804 (2)	0.59858 (9)	0.0624 (7)	
C24	0.88493 (13)	0.13378 (18)	0.48756 (11)	0.0628 (7)	
C25	0.93064 (11)	0.07611 (16)	0.41898 (9)	0.0476 (5)	
C26	1.00083 (12)	0.14359 (18)	0.38788 (10)	0.0566 (6)	
C27	1.04082 (13)	0.0836 (2)	0.32604 (11)	0.0674 (7)	
C28	1.01149 (16)	−0.0452 (2)	0.29468 (11)	0.0732 (8)	
C29	0.94303 (15)	−0.1147 (2)	0.32600 (12)	0.0724 (8)	
C30	0.90291 (12)	−0.05462 (18)	0.38750 (11)	0.0580 (6)	
H2	0.67140	0.84310	0.89410	0.0510*	
H6	0.40080	0.66260	0.80560	0.0590*	
H7	0.25700	0.66310	0.77640	0.0680*	
H8	0.19880	0.84970	0.70470	0.0680*	
H9	0.28570	1.03300	0.65900	0.0680*	
H10	0.42970	1.03080	0.68620	0.0580*	
H12	0.57100	0.65790	0.95460	0.0600*	
H13	0.48820	0.63440	1.06120	0.0710*	
H14	0.37480	0.79040	1.08000	0.0730*	
H15	0.34520	0.97260	0.99360	0.0650*	
H16	0.42670	0.99610	0.88650	0.0540*	
H17A	0.74780	0.93030	0.64210	0.0590*	
H17B	0.66620	0.87120	0.59990	0.0590*	
H18A	0.70700	0.62870	0.64390	0.0580*	
H18B	0.79480	0.69770	0.66800	0.0580*	
H20A	0.92760	0.56750	0.46210	0.0580*	
H20B	0.83690	0.60290	0.42820	0.0580*	
H21A	0.79410	0.36130	0.44560	0.0580*	
H21B	0.87760	0.36230	0.39730	0.0580*	
H22A	0.88930	0.26060	0.61530	0.0750*	
H22B	0.80110	0.29940	0.57750	0.0750*	
H23A	0.84760	0.49950	0.64700	0.0750*	
H23B	0.93450	0.50130	0.60350	0.0750*	
H24A	0.82440	0.11510	0.48110	0.0750*	
H24B	0.90420	0.07860	0.53140	0.0750*	
H26	1.02140	0.23040	0.40880	0.0680*	
H27	1.08790	0.13050	0.30540	0.0810*	
H28	1.03800	−0.08480	0.25250	0.0880*	
H29	0.92360	−0.20280	0.30560	0.0870*	
H30	0.85630	−0.10270	0.40830	0.0700*	

### Atomic displacement parameters (Å^2^)

**Table:**

	*U*^11^	*U*^22^	*U*^33^	*U*^12^	*U*^13^	*U*^23^
O1	0.0356 (7)	0.0821 (8)	0.0516 (6)	0.0029 (6)	0.0057 (5)	−0.0074 (6)
O2	0.0572 (8)	0.0642 (7)	0.0423 (6)	0.0168 (6)	0.0084 (5)	−0.0007 (5)
N1	0.0377 (7)	0.0554 (7)	0.0374 (6)	0.0085 (6)	0.0074 (5)	−0.0026 (6)
N2	0.0392 (7)	0.0508 (7)	0.0386 (7)	0.0076 (6)	0.0029 (6)	−0.0026 (5)
N3	0.0500 (8)	0.0562 (7)	0.0388 (7)	0.0133 (6)	0.0059 (6)	−0.0037 (6)
N4	0.0533 (9)	0.0515 (7)	0.0488 (8)	0.0122 (6)	0.0070 (6)	0.0015 (6)
C1	0.0388 (9)	0.0487 (8)	0.0416 (8)	0.0071 (7)	0.0022 (7)	−0.0051 (7)
C2	0.0416 (9)	0.0529 (8)	0.0341 (7)	0.0067 (7)	0.0008 (6)	−0.0043 (6)
C3	0.0402 (9)	0.0367 (7)	0.0371 (7)	0.0055 (6)	0.0033 (6)	−0.0021 (6)
C4	0.0396 (8)	0.0372 (7)	0.0366 (8)	0.0051 (6)	0.0037 (6)	−0.0012 (6)
C5	0.0388 (8)	0.0420 (7)	0.0342 (7)	0.0034 (6)	0.0022 (6)	−0.0048 (6)
C6	0.0466 (10)	0.0452 (8)	0.0551 (9)	0.0012 (7)	−0.0026 (7)	0.0055 (7)
C7	0.0461 (10)	0.0561 (10)	0.0683 (11)	−0.0091 (8)	0.0007 (8)	0.0012 (9)
C8	0.0397 (10)	0.0632 (10)	0.0674 (11)	0.0031 (8)	−0.0076 (8)	−0.0086 (9)
C9	0.0513 (11)	0.0553 (9)	0.0640 (11)	0.0079 (8)	−0.0126 (9)	0.0052 (8)
C10	0.0446 (9)	0.0486 (9)	0.0509 (9)	0.0012 (7)	−0.0028 (7)	0.0039 (7)
C11	0.0379 (8)	0.0458 (8)	0.0337 (7)	0.0012 (6)	0.0020 (6)	−0.0024 (6)
C12	0.0521 (10)	0.0534 (9)	0.0440 (9)	0.0074 (8)	0.0029 (7)	0.0014 (7)
C13	0.0657 (12)	0.0676 (10)	0.0441 (9)	0.0003 (9)	0.0070 (8)	0.0124 (8)
C14	0.0550 (11)	0.0813 (12)	0.0455 (9)	−0.0051 (10)	0.0153 (8)	0.0001 (9)
C15	0.0413 (10)	0.0713 (10)	0.0489 (9)	0.0076 (8)	0.0083 (8)	−0.0077 (8)
C16	0.0421 (9)	0.0524 (8)	0.0417 (8)	0.0069 (7)	0.0016 (7)	−0.0003 (7)
C17	0.0487 (10)	0.0600 (9)	0.0385 (8)	0.0092 (8)	0.0117 (7)	−0.0013 (7)
C18	0.0501 (10)	0.0519 (9)	0.0426 (8)	0.0015 (7)	0.0098 (7)	−0.0071 (7)
C19	0.0350 (8)	0.0467 (8)	0.0413 (8)	−0.0018 (6)	0.0038 (6)	−0.0056 (7)
C20	0.0472 (10)	0.0557 (9)	0.0432 (8)	0.0099 (7)	0.0115 (7)	−0.0024 (7)
C21	0.0462 (10)	0.0566 (9)	0.0427 (8)	0.0080 (7)	0.0054 (7)	−0.0028 (7)
C22	0.0721 (13)	0.0687 (11)	0.0465 (9)	0.0236 (9)	0.0087 (9)	0.0087 (8)
C23	0.0660 (13)	0.0771 (12)	0.0439 (9)	0.0254 (10)	−0.0028 (8)	−0.0051 (8)
C24	0.0664 (12)	0.0523 (10)	0.0699 (12)	0.0077 (8)	0.0195 (10)	0.0062 (9)
C25	0.0438 (9)	0.0430 (8)	0.0560 (9)	0.0066 (7)	0.0039 (7)	0.0052 (7)
C26	0.0504 (11)	0.0491 (9)	0.0704 (11)	−0.0015 (8)	0.0078 (9)	−0.0006 (8)
C27	0.0567 (12)	0.0738 (12)	0.0721 (12)	0.0094 (10)	0.0182 (10)	0.0148 (10)
C28	0.0857 (16)	0.0784 (13)	0.0557 (11)	0.0254 (12)	0.0058 (11)	−0.0058 (10)
C29	0.0785 (16)	0.0632 (11)	0.0754 (13)	0.0039 (10)	−0.0073 (12)	−0.0143 (10)
C30	0.0475 (11)	0.0512 (9)	0.0751 (12)	−0.0004 (8)	−0.0013 (9)	0.0040 (9)

### Geometric parameters (Å, °)

**Table:**

O1—C1	1.2263 (19)	C25—C30	1.383 (2)
O2—C19	1.2232 (18)	C26—C27	1.378 (3)
N1—N2	1.3527 (18)	C27—C28	1.375 (3)
N1—C1	1.3832 (19)	C28—C29	1.368 (3)
N1—C17	1.468 (2)	C29—C30	1.375 (3)
N2—C4	1.3064 (19)	C2—H2	0.9300
N3—C19	1.343 (2)	C6—H6	0.9300
N3—C20	1.4655 (19)	C7—H7	0.9300
N3—C23	1.459 (2)	C8—H8	0.9300
N4—C21	1.462 (2)	C9—H9	0.9300
N4—C22	1.462 (2)	C10—H10	0.9300
N4—C24	1.460 (2)	C12—H12	0.9300
C1—C2	1.435 (2)	C13—H13	0.9300
C2—C3	1.352 (2)	C14—H14	0.9300
C3—C4	1.441 (2)	C15—H15	0.9300
C3—C11	1.488 (2)	C16—H16	0.9300
C4—C5	1.487 (2)	C17—H17A	0.9700
C5—C6	1.387 (2)	C17—H17B	0.9700
C5—C10	1.378 (2)	C18—H18A	0.9700
C6—C7	1.383 (3)	C18—H18B	0.9700
C7—C8	1.374 (2)	C20—H20A	0.9700
C8—C9	1.375 (2)	C20—H20B	0.9700
C9—C10	1.381 (3)	C21—H21A	0.9700
C11—C12	1.384 (2)	C21—H21B	0.9700
C11—C16	1.388 (2)	C22—H22A	0.9700
C12—C13	1.383 (2)	C22—H22B	0.9700
C13—C14	1.374 (3)	C23—H23A	0.9700
C14—C15	1.371 (2)	C23—H23B	0.9700
C15—C16	1.379 (2)	C24—H24A	0.9700
C17—C18	1.514 (2)	C24—H24B	0.9700
C18—C19	1.516 (2)	C26—H26	0.9300
C20—C21	1.507 (2)	C27—H27	0.9300
C22—C23	1.501 (3)	C28—H28	0.9300
C24—C25	1.509 (3)	C29—H29	0.9300
C25—C26	1.379 (2)	C30—H30	0.9300
			
O1···C18	3.0755 (18)	H6···N2^vii^	2.8500
O2···C2^i^	3.3470 (19)	H7···C1^vii^	3.0400
O1···H17A	2.6000	H7···H17A^vii^	2.5700
O1···H27^ii^	2.6200	H8···C25^ix^	3.0400
O1···H20B^iii^	2.7200	H8···C30^ix^	2.9400
O1···H29^iv^	2.7400	H9···C2^vi^	3.0500
O1···H18B	2.5600	H9···C30^ix^	3.0700
O2···H20B	2.3500	H9···H30^ix^	2.5900
O2···H30^v^	2.6200	H10···N2	2.7600
O2···H17B	2.4400	H10···C3^vi^	2.8800
O2···H2^i^	2.4700	H12···C2	2.8400
N3···N4	2.8564 (18)	H12···H2	2.5500
N4···N3	2.8564 (18)	H13···N2^iii^	2.8400
N1···H6^vi^	2.9400	H13···C10^iii^	3.0600
N2···H13^i^	2.8400	H15···C19^vi^	2.8700
N2···H10	2.7600	H16···C4	2.9700
N2···H6^vi^	2.8500	H16···C5	2.7900
N4···H26	2.6400	H16···H18A^vi^	2.4700
C1···C7^vi^	3.536 (2)	H17A···O1	2.6000
C2···O2^iii^	3.3470 (19)	H17A···H7^vi^	2.5700
C5···C16	3.128 (2)	H17B···O2	2.4400
C6···C16	3.215 (2)	H18A···C23	3.0600
C6···C11	3.187 (2)	H18A···H23A	2.5000
C7···C1^vii^	3.536 (2)	H18A···H16^vii^	2.4700
C11···C6	3.187 (2)	H18B···O1	2.5600
C11···C15^viii^	3.441 (2)	H18B···C1	2.9000
C14···C16^viii^	3.548 (2)	H18B···C23	2.6500
C15···C16^viii^	3.484 (2)	H18B···H23A	2.0200
C15···C11^viii^	3.441 (2)	H18B···H27^ii^	2.4600
C16···C5	3.128 (2)	H20A···H23B	2.5700
C16···C15^viii^	3.484 (2)	H20A···H23B^ii^	2.5400
C16···C14^viii^	3.548 (2)	H20B···O2	2.3500
C16···C6	3.215 (2)	H20B···O1^i^	2.7200
C18···O1	3.0755 (18)	H21A···H22B	2.4000
C21···C26	3.339 (2)	H21A···H24A	2.3800
C26···C21	3.339 (2)	H21B···C25	2.7600
C1···H18B	2.9000	H21B···C26	2.7800
C1···H7^vi^	3.0400	H21B···H26	2.5600
C2···H12	2.8400	H22A···H24B	2.2400
C2···H9^vii^	3.0500	H22A···C28^x^	2.9600
C3···H10^vii^	2.8800	H22B···H21A	2.4000
C3···H6	3.0500	H22B···H24A	2.4200
C4···H16	2.9700	H23A···C18	2.4600
C5···H16	2.7900	H23A···H18A	2.5000
C10···H13^i^	3.0600	H23A···H18B	2.0200
C11···H6	2.8800	H23B···H20A	2.5700
C12···H2	2.8300	H23B···H20A^ii^	2.5400
C18···H23A	2.4600	H23B···H26^ii^	2.5500
C19···H15^vii^	2.8700	H24A···H21A	2.3800
C21···H26	3.0400	H24A···H22B	2.4200
C23···H18A	3.0600	H24A···H30	2.4200
C23···H18B	2.6500	H24B···H22A	2.2400
C25···H8^ix^	3.0400	H24B···C25^x^	3.0700
C25···H24B^x^	3.0700	H24B···C26^x^	2.8800
C25···H21B	2.7600	H24B···C27^x^	3.0400
C26···H21B	2.7800	H26···N4	2.6400
C26···H24B^x^	2.8800	H26···C21	3.0400
C27···H29^xi^	3.0900	H26···H21B	2.5600
C27···H24B^x^	3.0400	H26···H23B^ii^	2.5500
C28···H22A^x^	2.9600	H27···H29^xi^	2.4900
C30···H8^ix^	2.9400	H27···O1^ii^	2.6200
C30···H9^ix^	3.0700	H27···H18B^ii^	2.4600
H2···C12	2.8300	H29···C27^xii^	3.0900
H2···H12	2.5500	H29···H27^xii^	2.4900
H2···O2^iii^	2.4700	H29···O1^xiii^	2.7400
H6···C3	3.0500	H30···O2^xiv^	2.6200
H6···C11	2.8800	H30···H24A	2.4200
H6···N1^vii^	2.9400	H30···H9^ix^	2.5900
			
N2—N1—C1	125.43 (12)	C9—C8—H8	120.00
N2—N1—C17	114.57 (12)	C8—C9—H9	120.00
C1—N1—C17	119.91 (13)	C10—C9—H9	120.00
N1—N2—C4	118.95 (12)	C5—C10—H10	119.00
C19—N3—C20	120.92 (13)	C9—C10—H10	119.00
C19—N3—C23	126.61 (13)	C11—C12—H12	120.00
C20—N3—C23	112.46 (13)	C13—C12—H12	120.00
C21—N4—C22	108.86 (13)	C12—C13—H13	120.00
C21—N4—C24	112.48 (13)	C14—C13—H13	120.00
C22—N4—C24	108.60 (13)	C13—C14—H14	120.00
O1—C1—N1	120.27 (13)	C15—C14—H14	120.00
O1—C1—C2	125.99 (14)	C14—C15—H15	120.00
N1—C1—C2	113.74 (13)	C16—C15—H15	120.00
C1—C2—C3	122.58 (13)	C11—C16—H16	120.00
C2—C3—C4	117.35 (13)	C15—C16—H16	120.00
C2—C3—C11	120.70 (13)	N1—C17—H17A	110.00
C4—C3—C11	121.94 (12)	N1—C17—H17B	110.00
N2—C4—C3	121.88 (13)	C18—C17—H17A	110.00
N2—C4—C5	114.25 (12)	C18—C17—H17B	110.00
C3—C4—C5	123.87 (13)	H17A—C17—H17B	108.00
C4—C5—C6	121.83 (13)	C17—C18—H18A	109.00
C4—C5—C10	119.70 (13)	C17—C18—H18B	109.00
C6—C5—C10	118.46 (14)	C19—C18—H18A	109.00
C5—C6—C7	120.37 (15)	C19—C18—H18B	109.00
C6—C7—C8	120.49 (16)	H18A—C18—H18B	108.00
C7—C8—C9	119.50 (17)	N3—C20—H20A	110.00
C8—C9—C10	120.06 (16)	N3—C20—H20B	110.00
C5—C10—C9	121.11 (15)	C21—C20—H20A	110.00
C3—C11—C12	120.00 (13)	C21—C20—H20B	110.00
C3—C11—C16	121.25 (12)	H20A—C20—H20B	108.00
C12—C11—C16	118.73 (14)	N4—C21—H21A	109.00
C11—C12—C13	120.49 (15)	N4—C21—H21B	109.00
C12—C13—C14	120.02 (16)	C20—C21—H21A	109.00
C13—C14—C15	120.12 (16)	C20—C21—H21B	109.00
C14—C15—C16	120.13 (16)	H21A—C21—H21B	108.00
C11—C16—C15	120.50 (14)	N4—C22—H22A	109.00
N1—C17—C18	110.48 (12)	N4—C22—H22B	109.00
C17—C18—C19	111.68 (12)	C23—C22—H22A	109.00
O2—C19—N3	122.30 (14)	C23—C22—H22B	109.00
O2—C19—C18	120.13 (14)	H22A—C22—H22B	108.00
N3—C19—C18	117.52 (13)	N3—C23—H23A	110.00
N3—C20—C21	110.24 (13)	N3—C23—H23B	110.00
N4—C21—C20	111.18 (13)	C22—C23—H23A	110.00
N4—C22—C23	111.52 (14)	C22—C23—H23B	110.00
N3—C23—C22	110.54 (14)	H23A—C23—H23B	108.00
N4—C24—C25	115.53 (14)	N4—C24—H24A	108.00
C24—C25—C26	123.48 (15)	N4—C24—H24B	108.00
C24—C25—C30	118.26 (15)	C25—C24—H24A	108.00
C26—C25—C30	118.20 (16)	C25—C24—H24B	108.00
C25—C26—C27	120.65 (16)	H24A—C24—H24B	107.00
C26—C27—C28	120.36 (19)	C25—C26—H26	120.00
C27—C28—C29	119.52 (19)	C27—C26—H26	120.00
C28—C29—C30	120.14 (18)	C26—C27—H27	120.00
C25—C30—C29	121.12 (17)	C28—C27—H27	120.00
C1—C2—H2	119.00	C27—C28—H28	120.00
C3—C2—H2	119.00	C29—C28—H28	120.00
C5—C6—H6	120.00	C28—C29—H29	120.00
C7—C6—H6	120.00	C30—C29—H29	120.00
C6—C7—H7	120.00	C25—C30—H30	119.00
C8—C7—H7	120.00	C29—C30—H30	119.00
C7—C8—H8	120.00		
			
C1—N1—N2—C4	−1.0 (2)	C3—C4—C5—C10	126.30 (15)
C17—N1—N2—C4	175.45 (13)	N2—C4—C5—C10	−54.31 (18)
N2—N1—C1—O1	−177.67 (14)	N2—C4—C5—C6	124.74 (15)
C17—N1—C1—O1	6.0 (2)	C3—C4—C5—C6	−54.7 (2)
N2—N1—C1—C2	2.7 (2)	C6—C5—C10—C9	0.4 (2)
C17—N1—C1—C2	−173.61 (13)	C10—C5—C6—C7	0.2 (2)
N2—N1—C17—C18	−107.77 (14)	C4—C5—C10—C9	179.48 (15)
C1—N1—C17—C18	68.93 (17)	C4—C5—C6—C7	−178.86 (14)
N1—N2—C4—C3	−1.5 (2)	C5—C6—C7—C8	−0.9 (3)
N1—N2—C4—C5	179.11 (12)	C6—C7—C8—C9	1.0 (3)
C20—N3—C19—O2	−0.7 (2)	C7—C8—C9—C10	−0.4 (3)
C23—N3—C19—O2	178.20 (16)	C8—C9—C10—C5	−0.3 (3)
C23—N3—C20—C21	53.97 (18)	C12—C11—C16—C15	−1.2 (2)
C19—N3—C20—C21	−127.01 (15)	C3—C11—C16—C15	−179.40 (14)
C23—N3—C19—C18	−4.4 (2)	C3—C11—C12—C13	179.70 (15)
C19—N3—C23—C22	127.24 (17)	C16—C11—C12—C13	1.5 (2)
C20—N3—C19—C18	176.74 (14)	C11—C12—C13—C14	−0.6 (3)
C20—N3—C23—C22	−53.8 (2)	C12—C13—C14—C15	−0.6 (3)
C24—N4—C21—C20	179.51 (14)	C13—C14—C15—C16	0.9 (3)
C21—N4—C22—C23	−58.98 (19)	C14—C15—C16—C11	0.0 (3)
C22—N4—C21—C20	59.10 (17)	N1—C17—C18—C19	167.27 (13)
C24—N4—C22—C23	178.24 (16)	C17—C18—C19—N3	164.55 (14)
C22—N4—C24—C25	−170.09 (16)	C17—C18—C19—O2	−18.0 (2)
C21—N4—C24—C25	69.35 (19)	N3—C20—C21—N4	−56.76 (18)
N1—C1—C2—C3	−2.0 (2)	N4—C22—C23—N3	56.5 (2)
O1—C1—C2—C3	178.38 (15)	N4—C24—C25—C26	22.2 (2)
C1—C2—C3—C11	178.92 (13)	N4—C24—C25—C30	−160.63 (16)
C1—C2—C3—C4	−0.1 (2)	C24—C25—C26—C27	178.65 (17)
C11—C3—C4—C5	2.3 (2)	C30—C25—C26—C27	1.4 (3)
C2—C3—C4—C5	−178.64 (13)	C24—C25—C30—C29	−178.55 (18)
C2—C3—C11—C12	−49.2 (2)	C26—C25—C30—C29	−1.2 (3)
C2—C3—C11—C16	128.97 (15)	C25—C26—C27—C28	−0.4 (3)
C4—C3—C11—C12	129.80 (15)	C26—C27—C28—C29	−1.0 (3)
C4—C3—C11—C16	−52.04 (19)	C27—C28—C29—C30	1.2 (3)
C11—C3—C4—N2	−177.02 (13)	C28—C29—C30—C25	−0.1 (3)
C2—C3—C4—N2	2.0 (2)		

Symmetry codes: (i) *x*, −*y*+3/2, *z*−1/2; (ii) −*x*+2, −*y*+1, −*z*+1; (iii) *x*, −*y*+3/2, *z*+1/2; (iv) *x*, −*y*+1/2, *z*+1/2; (v) *x*, *y*+1, *z*; (vi) −*x*+1, *y*+1/2, −*z*+3/2; (vii) −*x*+1, *y*−1/2, −*z*+3/2; (viii) −*x*+1, −*y*+2, −*z*+2; (ix) −*x*+1, −*y*+1, −*z*+1; (x) −*x*+2, −*y*, −*z*+1; (xi) −*x*+2, *y*+1/2, −*z*+1/2; (xii) −*x*+2, *y*−1/2, −*z*+1/2; (xiii) *x*, −*y*+1/2, *z*−1/2; (xiv) *x*, *y*−1, *z*.

### Hydrogen-bond geometry (Å, °)

**Table:**

*D*—H···*A*	*D*—H	H···*A*	*D*···*A*	*D*—H···*A*
C2—H2···O2^iii^	0.93	2.47	3.3470 (19)	157
C18—H18B···O1	0.97	2.56	3.0755 (18)	113
C20—H20B···O2	0.97	2.35	2.759 (2)	105

Symmetry codes: (iii) *x*, −*y*+3/2, *z*+1/2.
